# Enhanced Ciliogenesis of Human Bronchial Epithelial Cells by Simulated Microgravity

**DOI:** 10.3390/life15121864

**Published:** 2025-12-05

**Authors:** Seung Hyun Bang, Soyoung Hwang, Seon Young Choi, Hyun Joo Kim, Joo Hyung Kim, Sung Ho Lee, Jin Woo Lee, Kuk Hui Son

**Affiliations:** 1 Department of Health Sciences and Technology, GAIHST, Gachon University, 155, Gaetbeol-ro, Yeonsu-gu, Incheon 21999, Republic of Korea; 2 Department of Translational-Clinical Medicine, Gachon University, Incheon 21565, Republic of Korea; 3 Department of Thoracic and Cardiovascular Surgery, Gil Medical Center, College of Medicine, Gachon University, 21, Namdong-daero 774 Beon-gil, Namdong-gu, Incheon 21565, Republic of Korea; 4 Department of Mechanical Engineering, College of Engineering, Inha University, 100, Inha-ro, Michuhol-gu, Incheon 22212, Republic of Korea; 5 Department of Thoracic and Cardiovascular Surgery, Korea University Anam Hospital, Korea University College of Medicine, Seoul 08308, Republic of Korea; 6 Department of Molecular Medicine, College of Medicine, Gachon University, 155, Gaetbeol-ro, Yeonsu-gu, Incheon 21999, Republic of Korea

**Keywords:** ciliogenesis, microgravity, bronchial epithelial cell, cell cycle, cilia length

## Abstract

Spaceflight induces a wide array of effects on the human body, notably including pathological changes mediated by alterations in gravity. Abnormalities in the formation of primary cilia (ciliogenesis) can lead to cell cycle arrest and decreased epithelial cell proliferation, thereby delaying wound healing. To investigate the effect of microgravity on ciliogenesis in bronchial epithelial cells, we used a 3D clinostat to generate simulated microgravity (SMG) conditions. When BEAS-2B bronchial epithelial cells were exposed to SMG for 72 h, their proliferation was significantly reduced. The expression of Ki-67, which is not expressed in the G0 phase, decreased under SMG. Conversely, the expression of p27, which is expressed in the G0 and G1 phases, increased under SMG. These results suggest that SMG led to an increase in the number of cells in the quiescent phase. When the mRNA expressions of ARL13B (a marker of cilia assembly) and disassembly-related genes (Aurora A, NDE1, HDAC6, and DVL2) were evaluated, SMG upregulated ciliary assembly markers and downregulated disassembly markers. In addition, SMG increased the cilia length and number of ciliated cells. These findings suggest that SMG contributes to reduced cell proliferation through cell cycle arrest by disrupting normal ciliogenesis. Our findings indicate that SMG could delay lung injury by decreasing cell proliferation.

## 1. Introduction

Primary cilia are microtubule-based organelles that extend from the cell surface and function as cellular antennas. They sense and transmit environmental signals and play crucial roles in regulating important processes such as proliferation and differentiation [1,2,3]. Cells assemble primary cilia upon exiting mitosis (the M to G0/G1 phase), start to disassemble the primary cilium when re-entering the cell cycle (G1-S transition), and fully disassemble the primary cilium before entering mitosis (the G2 to M phase) [4]. During respiratory tract repair, primary cilia are observed in airway epithelial cells, suggesting their involvement in wound healing by modulating the cell cycle [5].

Cell proliferation is a cell division process that depends on the cell cycle. The cell cycle consists of an interphase (the G1, S, and G2 phases), during which the cell grows and prepares for division, and a mitotic phase (the M phase), during which actual cell division occurs [5,6,7,8,9]. When proliferation is not required, the cells enter a resting state known as the G0 phase. At specific checkpoints during the cycle, namely the G1/S checkpoint (entry into the cycle), G2/M checkpoint (DNA damage check), and spindle checkpoint (chromosome separation check), the cell checks internal or external signals whether to proceed or stop cell division. These checkpoints prevent abnormal or uncontrolled cell division, with the G1/S checkpoint being crucial for regulating proliferation [10,11,12,13]. Most cells remain in the G0 phase unless they are stimulated by proliferation signals, such as growth factors, hormones, cell cycle inhibitors, tumor suppressor genes, or mechanical stress [14,15,16,17].

Adult lung epithelial cells are mitotically quiescent under normal conditions. However, progenitor cells, such as basal or alveolar type 2 cells, can re-enter the cell cycle and divide or differentiate into other lung cell types during lung injury repair [18,19]. Ciliated cells are involved in bronchiolar epithelium repair by metaplasia and re-differentiation into columnar epithelial cells following bronchial injury [9]. Therefore, the proliferation of progenitor and ciliated cells is essential for rapid repair of the lung epithelium [20].

Simulated microgravity (SMG) affects various cellular responses by altering the ability of the cells to sense and respond to external stimuli [21]. Previous studies have shown that SMG reduces survival and increases apoptosis of bronchial epithelial cells (BEAS-2B) [21,22]. Similarly, the exposure of the lung adenocarcinoma cell line A549 to SMG results in cell cycle imbalance and growth inhibition [23]. Notably, SMG hinders cell cycle progression from G0/G1 to the S phase in endothelial-like EA.hy926 cells [24]. These findings suggest that SMG affects cell proliferation by modulating the cell cycle.

It is well established that cilia disassembly is required for cells to exit the G0 phase and enter the G1 phase of the cell cycle, and that microgravity can influence cell cycle progression. Based on this, it can be hypothesized that microgravity may affect cilia assembly or disassembly dynamics. However, there have been few studies addressing this topic directly. In this study, we hypothesized that SMG may interfere with cilia shortening (disassembly), thereby suppressing cell cycle entry and ultimately slowing cell proliferation. To test this, we exposed BEAS-2B cells to SMG and examined the effects on cilia dynamics and cell proliferation. We compared the proliferation of SMG-exposed cells and cells under normal gravity (NG). We also explored how SMG-induced changes in ciligenesis influence cell cycle entry, thereby elucidating the mechanisms underlying impaired wound repair in the respiratory epithelium under SMG.

## 2. Materials and Methods

### 2.1. Human Bronchial Epithelial Cell Culture

#### 2.1.1. Preparation of Humand Bronchial Epithelial Cell

BEAS-2B cells (KCLB, Seoul, Republic of Korea) were cultured in DMEM (high-glucose) (Welgene, Daegu, Republic of Korea) supplemented with 10% fetal bovine serum (FBS, Welgene, Daegu, Republic of Korea) and 1% penicillin/streptomycin (Gibco, Montana, MT, USA) at 37 °C under 5% CO_2_. Cells between passages 14 and 23 were used in the experiments. BEAS-2B cells have an approximate doubling time of 26 h under standard culture conditions.

#### 2.1.2. Serum Starvation

The BEAS-2B cells were seeded at 1 × 10^4^/cm^2^ in T-12.5 culture flasks with a filter (vent) cap (Biofil, Jet Bio-filtration, Guanzhou, China). The seeding density was optimized so that the cells reached approximately 90% confluence at the experimental endpoint. The cells were cultured in a high-glucose DMEM supplemented with 10% FBS and 1% penicillin–streptomycin for 24 h to allow cell attachment. Subsequently, the cells were then washed once with DPBS (Welgene, Daegu, Republic of Korea) to remove unattached cells and placed in serum-free high-glucose DMEM for 24 h to induce growth arrest.

To ensure that all BEAS-2B cells entered a uniform quiescent (G0) state before simulated microgravity (SMG) exposure, we first evaluated the optimal duration of serum starvation required to induce maximal primary cilia assembly. Because cells in the G0 state possess fully elongated primary cilia, we measured the expression of the cilia assembly marker ARL13B at multiple time points (0, 3, 6, 12, and 24 h) during serum deprivation. ARL13B expression progressively increased and reached its highest level at 24 h, indicating that cilia assembly—and therefore G0 synchronization—was maximal at this time point (Supplement Figure S1A). To confirm that serum starvation itself did not affect cell viability or proliferation during this period, we performed a CCK-8 assay and LIVE/DEAD imaging at 0 and 24 h. Both assays demonstrated no significant change in proliferative activity or cell death over 24 h of serum deprivation (Supplement Figure S1B,C), indicating that the starvation period synchronized the cell cycle without inducing cytotoxicity. Based on these findings, we set 24 h of serum starvation as the standardized preconditioning step prior to SMG exposure, ensuring that NG and SMG groups started from the same ciliated, non-proliferative state. Following 24 h incubation of serum deprivation, the medium was replaced to serum-containing high-glucose DMEM immediately before SMG exposure. This pretreatment enabled evaluation of cell proliferation changes under SMG [25].

### 2.2. Three-Dimensional (3D) Clinostat

**Figure 1:**
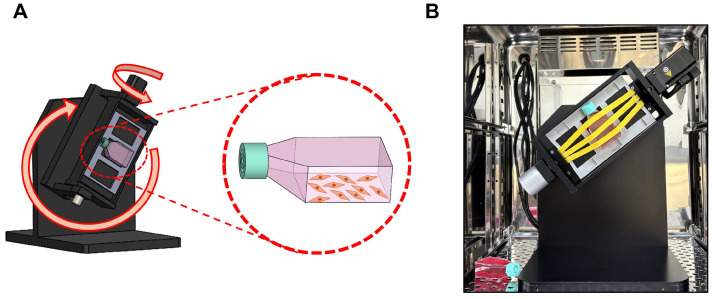
BEAS-2B cells exposure to SMG. (**A**) Schematic representation of the 3D clinostat used to generate simulated microgravity (SMG). The clinostat consists of two orthogonal rotation axes, enabling continuous randomization of the gravity vector. The orientation of the T-12.5 flask is indicated, with the cell-attached surface fixed against the stage frame to ensure complete medium coverage and elimination of air–liquid interfaces during rotation. (**B**) Photograph of the 3D clinostat installed inside a CO_2_ incubator during operation. T-12.5 flasks were completely filled with medium, and air bubbles were removed before rotation to prevent shear stress. Rotation was performed at 5 rpm for 24 or 72 h.

The 3D clinostat (version 2; Yonsei Academy of Science, Biophysics, and Medical Engineering Institute, Seoul, Republic of Korea) [26] was designed with two orthogonal rotation axes, enabling continuous changes in the direction and magnitude of gravity to simulate a microgravity (SMG) environment (Figure 1A,B). The rotation pattern, speed, and duration were controlled using software, and the device was rotated at 5 rpm for either 24 or 72 h. To validate that the 3D clinostat rotation at 5 rpm accurately generates a simulated microgravity environment, we performed SMG experiments at the same angular velocity using the same clinostat system in our previous work [27]. In that study, the microgravity condition produced by 5 rpm rotation was experimentally verified, and computational modeling demonstrated that the centrifugal acceleration experienced inside a T-flask (40.4 mm × 73.7 mm × 22.8 mm) ranged from approximately 0.0013 g at the flask edge to 0 g at the center, consistent with established SMG parameters.

BEAS-2B cells were seeded in T-12.5 culture flasks with filter cap and cultured for 24 h to allow cell adhesion. The vented caps contain a 0.22-µm hydrophobic membrane that allows passive gas exchange of O_2_ and CO_2_ while maintaining sterility, thereby supporting normal oxygenation even when the flasks are completely filled during clinorotation.

Before placing the flasks in the 3D clinostat, each flask was slowly filled with medium until no air bubbles remained, because trapped air can generate shear stress under rotation [28]. The same procedure—use of the identical filter-cap flasks, complete filling of medium, and removal of air bubbles—was also applied to the NG control group to ensure that the only experimental variable was exposure to simulated microgravity. Although a momentary shear force may occur at the boundary between the medium and the polystyrene wall when rotation begins, calculation based on the friction coefficient of polystyrene (0.5) indicated that the maximum wall shear stress at the flask edge was only 0.141 Pa, a level considered negligible for epithelial cells. This controlled setup allowed the observed SMG-induced effects to be attributed to microgravity rather than shear-related force.

For the experimental group (SMG), the flasks were positioned at the center of the 3D clinostat inside a CO_2_ incubator (5% CO_2_, 37 °C) and rotated at 5 rpm for either 24 or 72 h. The control group (normal gravity, NG) was cultured under static conditions in a 5% CO_2_ incubator without any movement after filling the medium to the same conditions as the experimental group.

### 2.3. Cell Viability in SMG

The cell viability following SMG exposure was assessed using the LIVE/DEAD™ Viability/Cytotoxicity Kit (Thermo Fisher Scientific, Waltham, MA, USA). LIVE/DEAD staining solution was prepared by mixing 2 mM ethidium homodimer-1 (EthD-1) and 5 mM Calcein AM in DPBS. After washing the SMG-exposed cells once with DPBS, each sample was treated with 1 mL of staining solution directly within the same T-12.5 culture flasks used for the experiment, without reseeding onto another culture plate and incubated at 37 °C in a 5% CO_2_ incubator for 30 min. Calcein AM staining was performed exclusively on live, non-fixed cells, as Calcein AM requires intact cell membranes and endogenous esterase activity to be converted into fluorescent Calcein; thus, the assay cannot be performed on fixed cells.

Importantly, the viability assay and imaging were performed directly in the same 12.5T flasks that were used for NG or SMG exposure, without reseeding or transferring cells to a separate imaging vessel. This prevented experimental disturbance of cell adhesion or viability status. Fluorescence microscopy (Eclipsets2, Nikon, Tokyo, Japan) was used to visualize the stained cells. Images were acquired using a 10× objective (N.A. 0.30). For each biological replicate, six regions of interest (ROI) were randomly selected within the same flask and analyzed. Cell viability analysis was conducted using three independent biological replicates (*n* = 3), each consisting of a separately cultured and independently SMG-exposed flask. Quantitative analysis was performed using the OCULAR software (Ver. 2.0, Teledyne Vision Solutions, Milpitas, CA, USA).

### 2.4. Cell Proliferation in SMG

The CCK-8 assay (Dojindo, Kumamoto, Japan) was used to measure cell proliferation following SMG exposure. Cells were washed once with DPBS, and treated 10% CCK-8 solution in culture medium directly within the same T-12.5 culture flasks used for the experiment, without reseeding onto another culture plate and incubated for 1 h at 37 °C in a 5% CO_2_ incubator under dark conditions. The absorbance was measured at 450 nm using a microplate reader (VERSAmax, Sunnyvale, CA, USA). Importantly, the cells were not detached or re-seeded into a separate microplate for this assay. Proliferation was assessed directly in the same 12.5T flasks that were used for NG or SMG exposure, without any intermediate transfer steps, in order to avoid disturbing cell adherence or altering proliferative behavior. All proliferation data were obtained from three independent biological replicates, each derived from separately cultured and individually SMG-exposed flasks.

For analysis, the absorbance of each sample (SMG and control groups at 0 h, 24 h, and 72 h) was calculated relative to the medium-only control. The resulting absorbance was then normalized to the corresponding 0 h value for each group.

### 2.5. Gene Expression Analysis

The mRNA expression was analyzed to investigate the effect of microgravity on cell proliferation, cell cycle, and primary cilia. p27 was used as an antiproliferative marker. E-cadherin was used as a cell junction marker. ADP-ribosylation factor-like protein 13 B (ARL13B, Bioneer, Daejeon, Republic of Korea) was used as a marker for ciliary assembly, whereas Aurora A, dishevelled segment polarity protein 2 (DVL2), histone deacetylase 6 (HDAC6), and nuclear distribution protein nudE homolog 1 (NDE1) (all from Bioneer, Daejeon, Republic of Korea) were used as markers of ciliary disassembly.

Following SMG exposure, the total RNA was extracted using RNAiso Plus (Takara Bio Inc., Shiga, Japan) and purified using the ReliaPrep RNA Cell Miniprep System (Promega, Madison, WI, USA). The RNA was quantified using a Nanodrop 2000 spectrophotometer (Thermo Fisher Scientific, Waltham, MA, USA), and complementary DNA (cDNA) was synthesized using the RevertAid H Minus First Strand cDNA Synthesis Kit (Thermo Fisher Scientific, Waltham, MA, USA). Real-time PCR was performed using TB Green^®^ Premix Ex Taq II (Tli RNaseH Plus) (Takara Bio Inc., Shiga, Japan). All of the primers listed in Table 1 were purchased from Bioneer (Daejeon, Republic of Korea). The relative gene expression levels were calculated using the 2^−^^ΔΔCt^ method, and all target genes were normalized to the housekeeping gene GAPDH. Each experiment was independently repeated three times.

### 2.6. Immunofluorescence

Following SMG exposure, each sample was washed once with DPBS and fixed with 4% paraformaldehyde (Biosesang, Gyeonggi-do, Republic of Korea) and incubated for 20 min. After three washes with DPBS (5 min each), the cells were permeabilized with 0.2% Triton X-100 (Sigma-Aldrich, Schnelldorf, Germany). Blocking was performed using 2% bovine serum albumin (BSA, Sigma-Aldrich, Schnelldorf, Germany) at room temperature for 1 h in the dark.

The samples were then incubated at 4 °C for 24 h with Ki-67 (Invitrogen, Carlsbad, CA, USA) at a 1:400 dilution in a primary antibody binding buffer containing 1% BSA and 0.2% Triton X-100. After three washes with DPBS containing 0.2% Triton X-100, the samples were incubated with goat anti-rabbit IgG (H+L) secondary antibody (Invitrogen, Carlsbad, CA, USA) at a 1:500 dilution for 1 h at room temperature in the dark. After removing the secondary antibody, F-actin was visualized by incubating the cells with Alexa Fluor 488-conjugated phalloidin (1:200; Thermo Fisher Scientific, Waltham, MA, USA) for 30 min. After washing, the cells were stained with 4′,6-diamidino-2-phenylindole (DAPI, Sigma-Aldrich, Schnelldorf, Germany) at 1 μg/mL for 5 min. The slides were washed with distilled water, air-dried, and mounted with glycerol (Sigma-Aldrich, Schnelldorf, Germany).

Acetylated α-tubulin (Ac-tubulin, Sigma-Aldrich, Schnelldorf, Germany) was diluted 1:700 in the primary antibody binding buffer and applied to the samples. The samples were incubated at 4 °C in the dark for 24 h. Following incubation, the samples were washed thrice for 5 min each at room temperature using a washing buffer composed of DPBS and 0.2% Triton X-100. Goat anti-rabbit IgG (H+L) secondary antibody (anti-mouse, Invitrogen, Carlsbad, CA, USA) was diluted 1:500 in the secondary antibody-binding buffer and applied to the samples, followed by incubation at room temperature in the dark for 1 h. Following secondary antibody incubation, the samples were washed thrice with a washing buffer for 5 min each to remove any unbound antibodies. DAPI was then applied at a concentration of 1 μg/mL and incubated for 5 min, followed by a single wash with triple-distilled water for 3 min. The samples were air-dried and mounted using glycerol.

All immunofluorescence staining and imaging were performed directly in the same 12.5T flasks used for NG or SMG exposure, without re-seeding the cells into another vessel. This approach minimized alterations in cell morphology and ciliary structure that could occur during additional handling. Fluorescent images were acquired using a 10× objective lens (numerical aperture 0.45) on a confocal laser-scanning microscope (LSM-710, ZEISS, Oberkochen, Germany). Each experiment was performed using three independent biological replicates (*n* = 3), and ten randomly selected ROIs were analyzed within each flask.

### 2.7. Cilia Length Measurement

Cells were fixed with 4% paraformaldehyde and permeabilized with 0.2% Triton X-100 in DPBS. To visualize the ciliary axoneme, cells were immunostained for acetylated α-tubulin and counterstained with DAPI to label nuclei. Fluorescence images were acquired using a confocal laser-scanning microscope equipped with a 10×/0.45 lens, under identical exposure settings for all samples.

For each experimental condition, 10 ROIs were captured per independent experiment, and all experiments were performed in triplicate (*n* = 3). Cilium length was measured in ImageJ V.1.54k (NIH, Bethesda, MD, USA) using the *Segmented Line* tool to trace each axoneme from the basal body to the distal tip. Only continuous acetylated α-tubulin-positive axonemes that were not truncated and measured ≥0.5 µm in length were included in the analysis.

### 2.8. Western Blot

Protein extracts were prepared from the NG and SMG groups by lysing them in RIPA buffer (Cell Signaling Technology, Danvers, MA, USA) containing a protease inhibitor cocktail (GenDEPOT, Baker, TX, USA) on ice for 20 min. The lysates were centrifuged at 12,000 rpm for 25 min at 4 °C, and the supernatants were collected. Protein concentration was determined using the Bradford assay (Bio-Rad, Hercules, CA, USA). A total of 15 μg of protein was loaded onto a 10% SDS-PAGE gel, separated by electrophoresis, and transferred to a polyvinylidene difluoride membrane (Millipore, Burlington, MA, USA). Membranes were blocked with 5% skim milk and incubated overnight at 4 °C with GAPDH (HRP-conjugate, 1:1000), PCNA (1:1000), Rb (1:500), pRb (1:1000), and Cyclin D1 (1:500) (all from Cell Signaling Technology). After washing, membranes were incubated with HRP-conjugated anti-rabbit IgG secondary antibodies (1:1000) for 1 h at room temperature. Protein bands were visualized using enhanced chemiluminescence (ECL; Thermo Fisher Scientific) and imaged with the iBright FL1000 imager (Invitrogen) in chemiluminescence mode using the automatic exposure setting. Band intensities were quantified using ImageJ (NIH), and relative protein expression was normalized to GAPDH.

### 2.9. Statistical Analysis

All experiments were repeated three times to confirm statistical significance. Prism 9 (GraphPad, Boston, MA, USA) was used for analyses. The statistical significance was assessed using the Mann–Whitney U test, with significance levels set at * *p* < 0.05, ** *p* < 0.01, *** *p* < 0.001.

## 3. Results

### 3.1. Changes in Cell Survival and Cell Proliferation Rate with SMG Exposure

**Figure 2:**
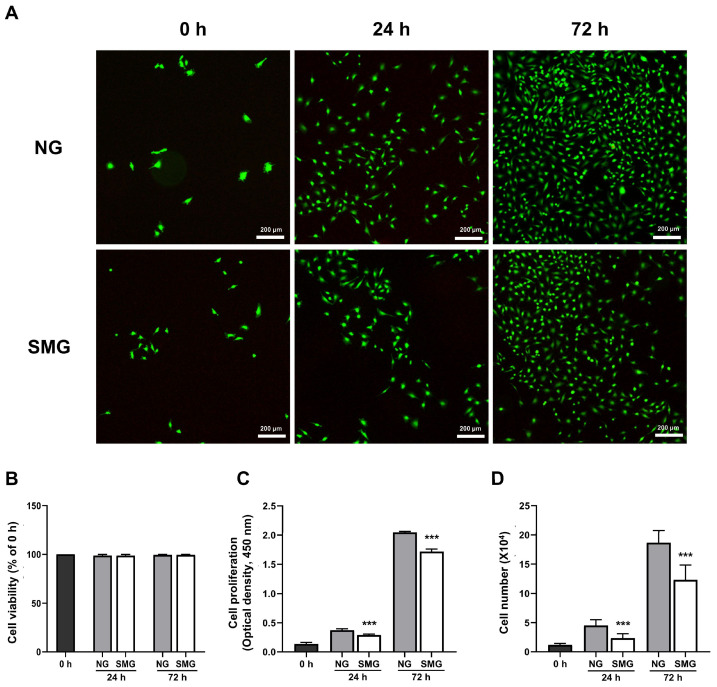
Cell viability and proliferation in response to SMG. (**A**) Representative LIVE/DEAD images of BEAS-2B cells under NG and SMG at 0, 24, and 72 h. Live cells are labeled with Calcein AM (green), which stains only viable, non-fixed cells with intact membranes, while dead cells are labeled with EthD-1 (red) (scale bar: 200 µm). (**B**) Quantitative analysis of cell viability expressed as the percentage of viable cells relative to the 0 h value for each condition. Viability remained above 90% in both NG and SMG groups across all time points (mean ± SD; *n* = 3). (**C**) Cell proliferation assessed using the CCK-8 assay at 0, 24, and 72 h under NG and SMG. Absorbance at 450 nm was background-corrected using a medium-only blank and normalized to the 0 h value. SMG significantly reduced proliferation at both 24 h and 72 h compared with NG (*** *p* < 0.001). (**D**) Total cell numbers measured using a hemocytometer at the indicated time points. SMG exposure resulted in significantly fewer cells compared with NG at 24 h and 72 h (*** *p* < 0.001), consistent with suppression of cell-cycle entry under microgravity. (NG; normal gravity, SMG; simulated microgravity) Error bars: mean ± SD; *n* = 3 biological replicates. Statistical significance was evaluated using the Mann–Whitney U test; * Compared with NG group, *** *p* < 0.001.

To investigate the impact of microgravity on bronchial epithelial cell survival, a LIVE/DEAD assay was performed. The results showed that dead cells were not observed in NG and SMG (Figure 2A,B). A CCK-8 assay was performed to evaluate the proliferation. As shown in Figure 2C, the proliferation rate of SMG cells was lower than that of NG cells at both 24 and 72 h. In addition, the number of counted cells in the SMG group was significantly lower than that in the NG group at 24 and 72 h (Figure 2D). Thus, SMG reduced the cell proliferation.

### 3.2. Change of Cell Cycle in Cells Exposed to SMG

The Ki-67 and p27 expression was evaluated to determine whether SMG affects the cell cycle. Ki-67 is expressed during all active phases of the cell cycle (G1, S, G2, and M) but not in quiescent (G0) cells, making it a reliable proliferation marker [29].

**Figure 3:**
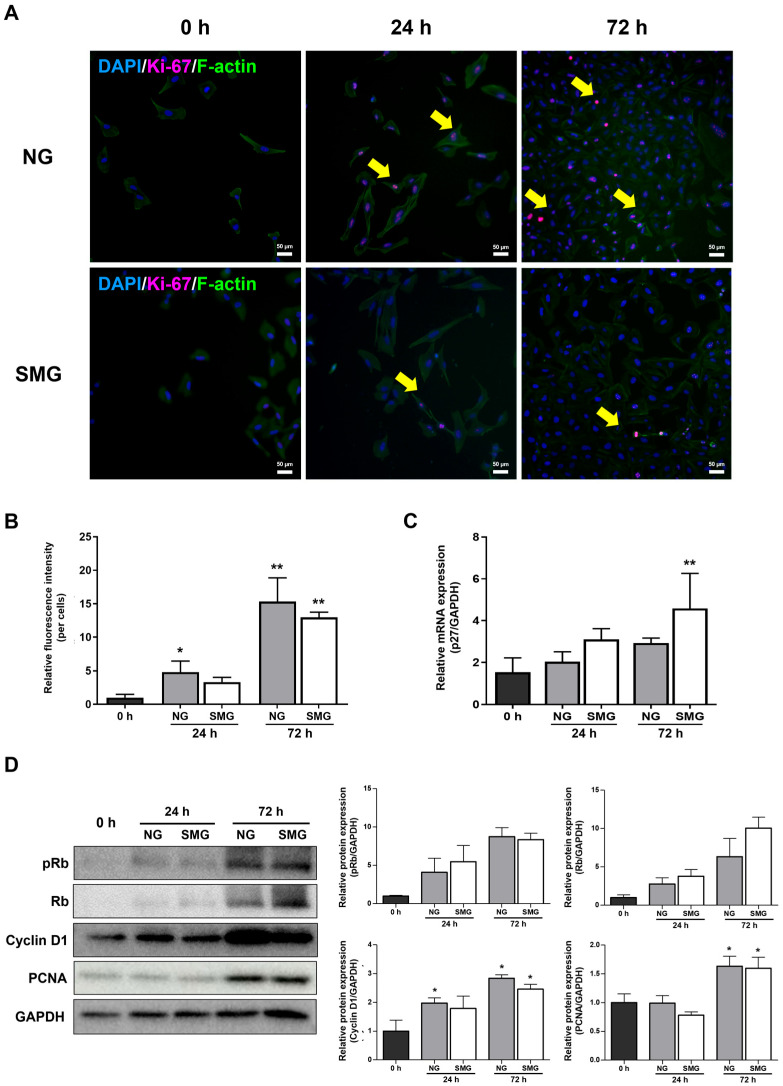
Cell cycle changes under SMG exposure. (**A**) Representative immunofluorescence images of BEAS-2B cells stained for Ki-67 (magenta) and DAPI (blue) under NG and SMG at 0, 24, and 72 h. Yellow arrows indicate Ki-67-positive nuclei (scale bar: 50 µm). (**B**) Quantification of Ki-67/F-actin fluorescence ratios. Ki-67 and F-actin were co-stained, and the ratio was calculated only from regions showing clear colocalization. Ten ROIs were analyzed per flask, with three biological replicates (*n* = 3). (**C**) Relative mRNA expression of p27 at 0, 24, and 72 h under NG and SMG, normalized to GAPDH using the 2^−ΔΔCt^ method. (**D**) Representative Western blots and quantification of G1/S cell-cycle regulators (Rb, pRb, Cyclin D1, PCNA) under NG and SMG at 24 and 72 h. GAPDH served as a loading control. (Error bars: mean ± SD; *n* = 3 biological replicates. Mann–Whitney U test; * Compared with NG group, * *p* < 0.05, ** *p* < 0.01).

Immunofluorescence analysis of Ki-67 and F-actin showed marked differences in cell-cycle activity between NG and SMG conditions. At 0 h, Ki-67-positive nuclei were rarely observed in either group (Figure 3A). At 24 h, Ki-67 expression increased in the NG group, whereas SMG-exposed cells displayed substantially fewer Ki-67-positive cells. By 72 h, the NG group exhibited a pronounced elevation in Ki-67 staining, while the SMG group maintained a markedly attenuated signal (Figure 3A). Quantitative analysis of the Ki-67/F-actin fluorescence ratio demonstrated a significant increase over time under NG conditions, whereas SMG consistently suppressed the ratio at both 24 h and 72 h (Figure 3B).

As a member of the universal cyclin-dependent kinase inhibitor family, p27 blocks the cell cycle at the G1/S phase checkpoint [30]. Thus, p27 is highly expressed in cells that are arrested in the G0 and G1 phases [30]. The expression of p27 mRNA was higher in the SMG group than in the NG group, indicating that progression into the S phase was inhibited under SMG (Figure 3C).

Consistent with these findings, Western blot analysis revealed lower levels of Rb phosphorylation, Cyclin D1 expression, and PCNA accumulation in SMG-exposed cells compared with NG (Figure 3D). Together, these imaging-based and biochemical measurements demonstrate that SMG markedly inhibits proliferation-associated signaling and prevents normal progression through the G1/S transition.

### 3.3. Change in Ciliogenesis Gene Expression Levels with SMG Exposure

**Figure 4:**
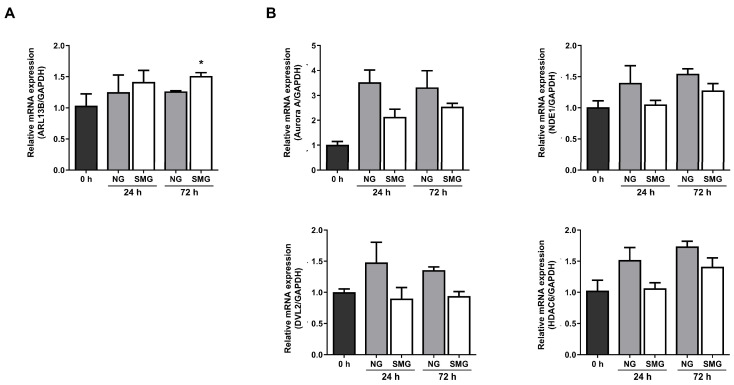
Analysis of changes in primary cilia ciliogenesis in response to SMG exposure. (**A**) Fold-change (2^−ΔΔCt^) in the cilia assembly marker ARL13B at 24 and 72 h under NG and SMG. (**B**) Fold-change in cilia disassembly markers Aurora A, NDE1, HDAC6, and DVL2 at 24 and 72 h under NG and SMG. (SMG; simulated microgravity group, NG; normal gravity group) *p*-values were calculated using the Mann–Whitney U test. * Compared with 0 h; * *p* < 0.05).

mRNA expression analysis was performed for ARL13B (a marker of cilia assembly) and Aurora A, NDE1, HDAC6, and DVL2 (disassembly-related genes) to explore whether SMG alters the primary cilia dynamics. At 24 h, ARL13B increased to 1.12-fold in NG and 1.41-fold in SMG relative to 0 h. At 72 h, ARL13B remained elevated (1.27-fold in NG and 1.46-fold in SMG), supporting a trend toward enhanced cilia assembly under SMG (Figure 4A).

Among the disassembly-related genes, Aurora A expression increased markedly in both groups at 24 h compared with 0 h, showing a 3.36-fold increase in NG and a 2.67-fold increase in SMG. At 72 h, Aurora A remained elevated (2.71-fold in NG, 2.66-fold in SMG), with the NG group showing a slightly greater induction (Figure 4B). NDE1 expression also increased over time, reaching 1.40-fold (NG) and 1.05-fold (SMG) at 24 h, and 1.55-fold (NG) and 1.28-fold (SMG) at 72 h. HDAC6 exhibited a similar pattern, increasing to 1.52-fold (NG) and 1.07-fold (SMG) at 24 h, and 1.74-fold (NG) and 1.39-fold (SMG) at 72 h. DVL2 expression remained relatively stable across all conditions, showing modest fluctuations without clear time- or gravity-dependent differences. Although most differences were not statistically significant in the analysis, the fold-change values consistently showed lower induction of disassembly-related genes in SMG compared with NG, whereas ARL13B showed a stronger increase under SMG. Collectively, these patterns indicate that SMG shifts the transcriptional balance toward ciliary assembly and retention rather than disassembly.

### 3.4. Change in Cilia Length with SMG Exposure

**Figure 5:**
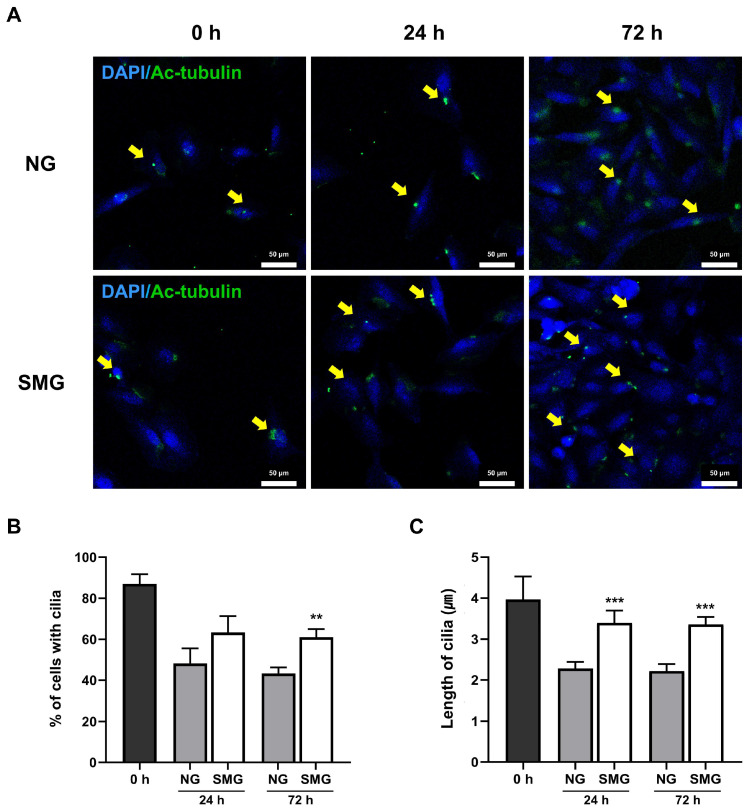
Alterations in primary cilia in response to SMG. (**A**) Representative immunofluorescence images of Ac-tubulin (green) and DAPI-stained nuclei (blue) in BEAS-2B cells under NG and SMG at 0, 24, and 72 h. Yellow arrows indicate primary cilia (scale bar: 50 µm). (**B**) Percentage of ciliated cells calculated as the number of Ac-tubulin-positive cells relative to total DAPI-positive nuclei per ROI. (**C**) Individual primary cilium length measurements (≥0.5 µm, continuous axonemes only), quantified using Image J Segmented Line tool. Ten ROIs were analyzed per experiment, with three biological replicates (*n* = 3). (SMG; simulated microgravity group, NG; normal gravity group) *p*-values were calculated using the Mann–Whitney U test. * Compared with NG group; ** *p* < 0.01, and *** *p* < 0.001.

Ac-tubulin immunofluorescence staining was performed to confirm whether SMG affects the length of primary cilia (Figure 5A). The ratio of ciliated cells was determined by calculating the number of Ac-tubulin-positive cells relative to the total number of DAPI-positive nuclei. This Ac-tubulin/DAPI ratio was consistently higher in the SMG group than in the NG group, although it gradually declined over time in both groups (Figure 5B).

The average cilia length was greater in SMG-exposed cells than in the NG group (Figure 5C).

## 4. Discussion

Tissue injury repair is initiated by the migration of inflammatory cells to the wound site and the removal of pathogens or tissue debris. Progenitors or cells such as fibroblasts, keratinocytes, and endothelial cells migrate to the wound site and begin to proliferate to repair the tissue [31]. These processes are influenced by gravitational changes. Skin wound healing is delayed during long-duration spaceflight [32]. In chronic wound lesions, fibroblasts are dysfunctional, which decreases the migration, proliferation, and synthesis of extracellular matrix processes [33,34,35,36]. These dysfunctional characteristics were also observed in fibroblasts exposed to SMG [37,38,39].

Gravity affects the lungs. During spaceflight, the alveolar volume and ventilation between the top and bottom of the lungs change [40,41]. Given its large surface area and exposure to air, the lung epithelium may be particularly vulnerable to gravity-related injuries [42]. Most previous studies investigating gravitational effects on the lung have focused on physiological changes such as lung volume, rather than examining how gravity directly affects epithelial cells. Therefore, in this study, we utilized bronchial epithelial cells—one of the major types of lung epithelial cells—to specifically investigate how SMG influences cell proliferation.

We used a 3D clinostat to generate the SMG environment. A clinorotation creates a centrifugal force that varies according to the rotational speed and distance of the sample from the rotation axis [43]. A 2D clinostat operates by rotating the samples continuously around the horizontal axis, inducing the gravity vector to change continuously with respect to the direction of the samples, which eventually constantly change their direction [43,44]. However, a 2D clinostat cannot completely remove the effect of gravity from the samples [45]. A 3D clinostat can rotate samples in three dimensions, creating more accurate SMG than a 2D clinostat [45].

The BEAS-2B cell line is an immortalized, non-tumorigenic human cell line that is obtained from a normal human bronchial epithelium of a non-cancerous individual [46]. BEAS-2B cells have been widely used as an in vitro model to evaluate respiratory diseases and toxicity [47]. Thus, we exposed BEAS-2B cells to SMG generated by a 3D clinostat. Exposure to SMG for up to 72 h did not induce cell death, as evaluated by the LIVE/DEAD assay. However, SMG exposure significantly decreased the cell proliferation rate.

In this study, we selected 24 h and 72 h as representative time points to capture the early and prolonged cellular responses to simulated microgravity. Prior work has shown that microgravity-induced changes in proliferation and cell-cycle regulators emerge gradually, with transient or unstable alterations occurring around 48 h, followed by stabilization by 72 h. Tan et al. reported that SMG-induced suppression of BEAS-2B proliferation becomes clearly detectable after 48 h and reaches a stable inhibitory phase by 72 h [48]. Given that 48 h corresponds to an intermediate adaptive phase rather than a definitive response window, focusing on 24 h and 72 h allowed us to distinctly compare the onset and establishment of SMG-induced cell-cycle arrest.

SMG is also involved in cell cycle changes, leading to decreased proliferation. SMG decreased the proliferation of hematopoietic stem and progenitor cells by blocking the cell cycle at the G1/S transition [49]. The proliferation of Chang liver cells (CCL-13) decreased after 72 h of SMG exposure [50]. Moreover, SMG increased the number of cells in the G0/G1 phase and decreased the number of cells in the G2/M and S phases [51]. Marrow mesenchymal stem cells exposed to SMG also showed increased numbers of cells in G1 phase [52]. In addition, SMG induced G2/M-phase arrest in U251 glioma cells [53].

Our findings are consistent with those of previous studies. Accompanied by decreased proliferation, Ki-67-expressing cells decreased after 72 h of exposure in the SMG group compared with those in the NG group. In contrast, p27 expression was higher in the SMG group than in the NG group after 72 h. These results suggest that SMG decreased the number of cells in the G1/S phase and increased the number of cells in the G0 phase. This interpretation is further supported by our Western blot analysis of key G1/S-regulatory proteins. SMG markedly reduced the phosphorylation of Rb (pRb), a critical step required for the release of E2F transcription factors and initiation of S-phase entry. Likewise, Cyclin D1, which drives progression through early G1, and PCNA, a core component of DNA replication machinery, were both substantially lower under SMG than NG. Together, these protein-level changes corroborate the Ki-67 and p27 results and demonstrate that SMG robustly suppresses G1/S transition, thereby maintaining a larger proportion of cells in a quiescent G0/G1 state.

The lengths of primary cilia change dynamically via assembly and disassembly, which are tightly linked to cell cycle progression [54]. Serum starvation induces cell cycle arrest and assembly of cilia, and cells remain in the G0/G1 phase [55]. In contrast, serum-supplemented media increase cilia disassembly [10,55]. DVL2 inhibits cilia biogenesis [56]. Phosphorylation of DVL2 stabilizes the human enhancer of filamentation one protein (HEF1/Cas-L/NEDD9), resulting in its binding to Aurora A kinase [55,57]. The HEF1/Aurora A kinase complex in the basal body leads to phosphorylation of HDAC6, which eventually activates HDAC6 [55,57]. HDAC6 induces the disassembly of cilia via deacetylation of Ac-tubulin [58]. This deacetylation leads to destabilization of axonemal microtubules and ciliary resorption [59,60,61]. The Aurora A/HDAC6 pathway involves ciliary disassembly during cell cycle re-entry and the inhibition of ciliary reassembly in proliferating cells [62]. NDE1 also decreases the cilia length by modulating dynein activity [63]. ARL13B, which is a small GTPase that localizes to the cilia, is involved in cilia length control [64]. Deletion of ARL13B leads to decreased cilia length [65].

Our results demonstrated that the ciliary assembly marker ARL13B increased under SMG compared with NG at both 24 h and 72 h, indicating a shift toward enhanced cilia assembly. In contrast, the induction of the ciliary disassembly markers Aurora A, NDE1, and HDAC6 was consistently lower in SMG than in NG, suggesting that SMG attenuates the transcriptional programs required for cilia resorption. These gene expression patterns (increased assembly and reduced disassembly signaling) align well with our imaging data, in which the proportion of ciliated cells was higher and the average cilium length was significantly longer in the SMG group than in the NG group. Together, these findings indicate that SMG shifts the dynamic balance of ciliary turnover toward retention and elongation, rather than shortening. These results suggest that SMG increased the number of cells in the G0 phase, which was associated with increased cilia length. As we evaluated only ciliary assembly and disassembly markers, only the exact mechanism of how SMG leads to increased cilia length was investigated in the present study. We suggest that increased assembly and decreased disassembly during SMG exposure resulted in increased cilia length. The fine mechanism by which SMG modulates cilia length should be evaluated in future studies. Although the precise mechanism by which SMG inhibits cilia shortening could not be elucidated in this study, our findings confirm that SMG-mediated inhibition of cilia shortening prevents cell cycle entry, consequently resulting in reduced proliferation.

Previous studies have shown that primary cilia are shortened by SMG, which is associated with decreased differentiation, maturation, and mineralization of rat calvarial osteoblasts [66,67]. These studies did not culture cells under serum starvation conditions before SMG exposure because they hypothesized that primary cilia function as sensors for bone metabolism [66,67]. In contrast, the primary objective of our study was to evaluate how SMG disrupts cell proliferation, a process tightly linked to the initiation of ciliogenesis. Thus, we employed a serum starvation-induced primary ciliogenesis model to ensure all cells were synchronized and ciliated in the G0 phase before SMG exposure. This crucial difference in the experimental model allowed us to isolate and observe how SMG exposure specifically impacts the dynamic equilibrium of ciliary length—a balance between assembly and disassembly.

However, it is important to acknowledge the limitations inherent in using the BEAS-2B model. We did not evaluate how microgravity affects ciliary control during wound healing. Specifically, the BEAS-2B cell line represents an intact, non-injured epithelium, which fundamentally differs from the environment of injured epithelium necessary for repair. In vivo lung injury repair is often mediated by progenitor cells, such as basal cells, which rapidly increase their proliferation rate upon damage. Our results, which demonstrate that SMG-induced changes in cilia length lead to decreased proliferation in quiescent cells, suggest that the effect of SMG may differ significantly between intact and injured tissue contexts. Therefore, our findings necessitate future studies utilizing a lung wound repair model to definitively conclude how SMG affects the required proliferative response and, consequently, lung tissue repair. Despite these model limitations, our study successfully elucidates a critical, basic regulatory axis—the cilia-cell cycle mechanism—that will be highly relevant for developing countermeasures to restore regenerative proliferation in microgravity environments.

## 5. Conclusions

BEAS-2B cells under SMG showed decreased proliferation, which was associated with an increased number of cells in the quiescent phase. SMG increased cilia assembly markers and decreased cilia disassembly markers, which were associated with an increased number of ciliated cells and increased cilia length. As SMG affects the wound healing process by decreasing cell proliferation, our results suggest that decreased proliferation owing to changes in the cell cycle and cilia could affect lung injury repair under SMG.

## Figures and Tables

**Table 1:** Primers for real-time PCR analysis.

Gene	Sequence (5′→3′)	T_m_ (°C)
GAPDH	F: ACCAGGTGGTCTCCTCTGAC	57
	R: TGCTTAGCCAAATTCGTTG	
ARL13B	F: AGCCTGTCAGGTTGGCAAAT	57
	R: ACGCTGCTCTGTTGTCTCTT	
Aurora A	F: AAGACTTGGGTCCTTGGGTC	57
	R: GTCCATGATGCCTCTAGCTGT	
HDAC6	F: GCCTCAATCACTGAGACATCC	57
	R: GGTGCCTTCTTGGTGACAACT	
DVL2	F: AGAGACAGCAGTGAGCATGG	57
	R: GGAATCTGTGACGCTGCTGA	
NDE1	F: GACACCATGCCACAAGGAGA	57
	R: TCCATGCGAAGGCGGTTATT	
p27	F: ATGTCAAACGTGCGAGTGTC	58
	R: TCTCTGCAGTGCTTCTCCAA	
E-cadherin	F: CATCTTTGTGCCTCCTGAAA	56
	R: TGGGCAGTGTAGGATGTGAT	

## Data Availability

The original contributions presented in this study are included in the article/Supplementary Material. Further inquiries can be directed to the corresponding authors.

## Supplementary Materials

The following supporting information can be downloaded at: https://www.mdpi.com/article/10.3390/life15121864/s1. Supplement Figure S1. Optimization and validation of 24 h serum starvation for G0 synchronization. Supplement Figure S2. Validation that SMG does not alter epithelial junction integrity (E-cadherin expression).

## Abbreviations

The following abbreviations are used in this manuscript:

NG	Normal gravity
SMG	Simulate microgravity
ARL13B	ADP-ribosylation factor-like protein 13B
NDE1	Nuclear distribution protein nudE homolog 1
HDAC6	Histone deacetylase 6
DVL2	Dishevelled segment polarity protein 2
Ac-tubulin	Acetylated α-tubulin
DAPI	4′,6-diamidino-2-phenylindole
