# Supplementary material for: Enhanced Ciliogenesis of Human Bronchial Epithelial Cells by Simulated Microgravity
Source: Life (Basel). 2025 Dec 5;15(12):1864. doi: 10.3390/life15121864 (PMC12735269; doi:10.3390/life15121864)
Supplement: Supplementary file 1 [file life-15-01864-s001.zip › life-3949351-supplementary.pdf]

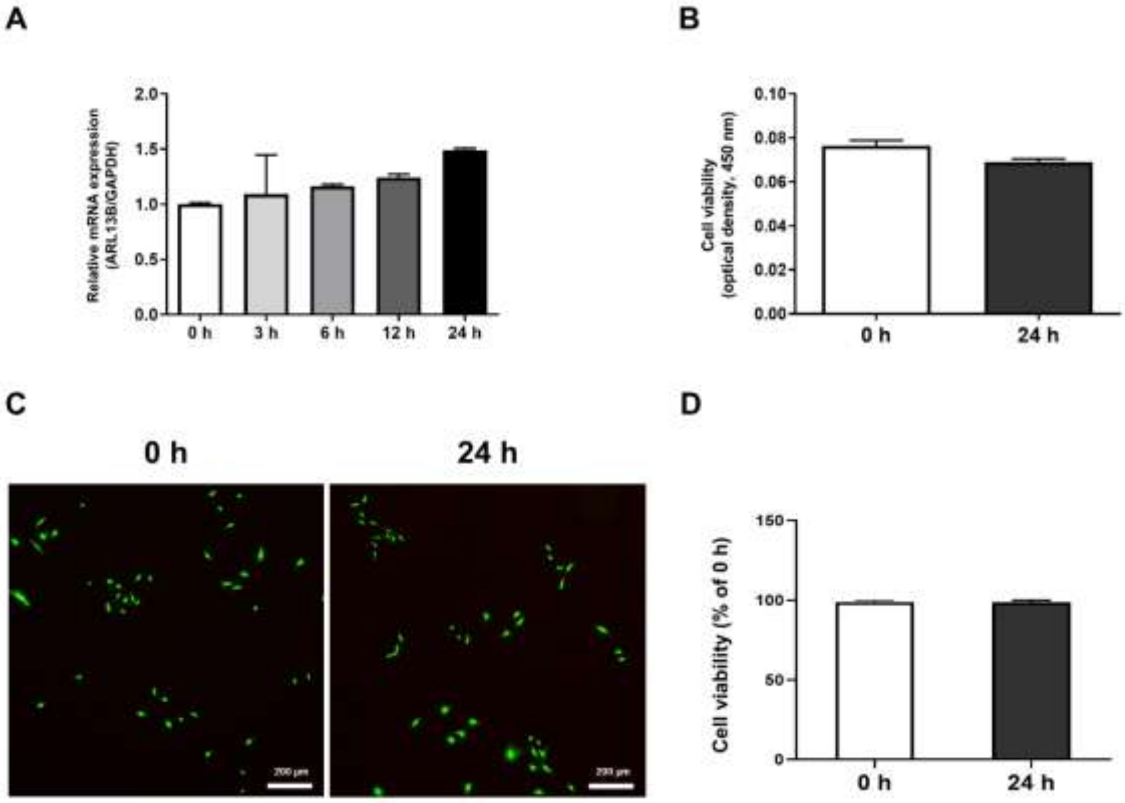

Supplement Figure S1. Optimization and validation of 24 h serum starvation for G0 synchronization (A) Time-dependent changes in ARL13B mRNA expression during serum starvation. BEAS-2B cells were cultured in serum-free medium for 0, 3, 6, 12, and 24 h. ARL13B expression gradually increased and reached its highest level at 24 h, indicating maximal primary cilia assembly and G0 synchronization ($2^{-\Delta\Delta Ct}$; mean ± SD; n = 3). (B) Cell proliferation measured by the CCK-8 assay at 0 h and 24 h of serum starvation. Absorbance at 450 nm showed no significant reduction after 24 h of serum deprivation, confirming that starvation did not negatively affect cell viability or proliferation capacity (mean ± SD; n = 3). (C) LIVE/DEAD imaging of BEAS-2B cells at 0 h and 24 h under serum-free conditions. Calcein AM (green) indicates live, non-fixed cells with intact membranes. No increase in dead cells was observed following 24 h of serum starvation (scale bar: 200 µm).

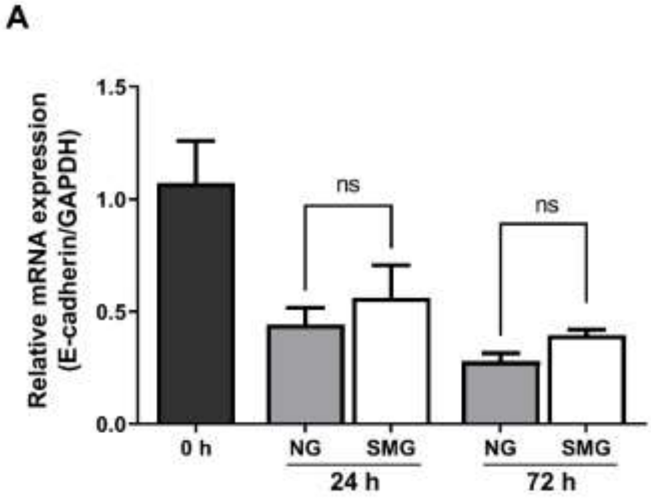

Supplement Figure S2. Validation that SMG does not alter epithelial junction integrity (E-cadherin expression). (A) Relative mRNA expression of E-cadherin at 0 h, 24 h, and 72 h under normal gravity (NG) and simulated microgravity (SMG) conditions. E-cadherin expression was measured by quantitative real-time PCR and normalized to GAPDH using the $2^{-\Delta\Delta Ct}$ method. No significant differences were observed between NG and SMG at either 24 h or 72 h, indicating that SMG did not alter epithelial junction integrity and that the experimental conditions did not induce shear-related disruption of epithelial phenotype (mean ± SD; n = 3; ns: not significant, Mann–Whitney U test).
